# circ2GO: A Database Linking Circular RNAs to Gene Function

**DOI:** 10.3390/cancers12102975

**Published:** 2020-10-14

**Authors:** Yanhong Lyu, Maiwen Caudron-Herger, Sven Diederichs

**Affiliations:** 1 Department of Thoracic Surgery, Division of Cancer Research, Medical Center-University of Freiburg, Faculty of Medicine, University of Freiburg, 79106 Freiburg, Germany; y.lyu@dkfz.de; 2 German Cancer Consortium (DKTK), Partner Site Freiburg, 79106 Freiburg, Germany; 3 Division of RNA Biology & Cancer, German Cancer Research Center (DKFZ), 69120 Heidelberg, Germany; m.caudron@dkfz.de

**Keywords:** circRNA, gene ontology, microRNA, lung cancer

## Abstract

**Simple Summary:**

Ribonucleic acids (RNAs) are generally linear chains of nucleotides which function in many cellular processes, best known in protein biosynthesis. In the last decade, circular RNAs have been discovered which are circularized after their synthesis and differ in important features from linear RNAs. These circular RNAs have meanwhile been implicated in important cellular processes in health and disease. Here, we present a comprehensive database, circ2GO, compiling and analyzing circular RNAs found in lung cancer cell lines providing the data in tables as well as visualizing it in transcript maps and in heatmaps. Importantly, we also provide easy-to-use online tools to find circular forms of genes associated with specific molecular functions, biological processes or cellular components or predict their targeted microRNAs. This resource will enable researchers to rapidly identify circular RNAs relevant for their specific research question.

**Abstract:**

Circular RNAs (circRNAs) play critical roles in a broad spectrum of physiological and pathological processes, including cancer. Here, we provide a comprehensive database—circ2GO—systematically linking circRNAs to the functions and processes of their linear counterparts. circ2GO contains 148,811 circular human RNAs originating from 12,251 genes, which we derived from deep transcriptomics after rRNA depletion in a panel of 60 lung cancer and non-transformed cell lines. The broad circRNA expression dataset is mapped to all isoforms of the respective gene. The data are visualized in transcript maps and in heatmaps, to intuitively display a comprehensive portrait for the abundance of circRNAs across transcripts and cell lines. By integrating gene ontology (GO) information for all genes in our dataset, circ2GO builds a connection between circRNAs and their host genes’ biological functions and molecular mechanisms. Additionally, circ2GO offers target predictions for circRNA—microRNA (miRNA) pairs for 25,166 highly abundant circRNAs from 6578 genes and 897 high-confidence human miRNAs. Visualization, user-friendliness, intuitive and advanced forward and reverse search options, batch processing and download options make circ2GO a comprehensive source for circRNA information to build hypotheses on their function, processes, and miRNA targets.

## 1. Introduction

Circular RNAs (circRNAs) constitute a class of single-stranded RNA with a covalent bond of the 3′-end to the 5′-end by back-splicing. The covalently closed continuous loop makes circRNAs resistant against degradation by exonucleases, and hence they have a longer half-life than their linear counterparts [1]. Growing evidence shows that circRNAs are widely expressed in vertebrate cells and show tissue-specific and cell type-specific expression patterns. circRNA biogenesis is believed to be regulated by signals *in cis*, as well as factors acting *in trans* to govern the context-dependent efficiency of circularization.

At the molecular level, circRNAs can affect the cell homeostasis, e.g., by interacting with RNA binding proteins (RBPs) [2], or by functioning as microRNA (miRNA) sponges [3,4]. Importantly, circRNAs are often intimately linked to the expression or function of their linear host gene [5,6,7,8]. Interestingly, recent studies found that some circRNAs encode functional peptides [9,10,11,12]. However, even though thousands of circRNAs have now been discovered, the underlying mechanisms regulating their biogenesis, function, degradation, and cellular localization remains unclear in most cases.

At the cellular level, circRNAs are important regulators in many cellular processes, such as cell signaling [13], embryonic development [14], cellular senescence [15], and control of the cell cycle [16]. They also play critical roles in the occurrence and development of various types of diseases [17], including cardiovascular diseases (e.g., atherosclerotic vascular disease risk [18,19]), neurological disorders (e.g., Alzheimer’s disease [20,21]), osteoarthritis [22], diabetes [23] and, most importantly, in cancer [24,25]. In malignant pathogenesis, circRNAs contribute to distinct human tumor entities including ovarian, prostate, liver, breast and lung cancers [26]. Lung cancer is one of the most fatal malignant diseases in the world. According to the Global Cancer Observatory (GCO) in 2018, 11.6% of total cancer cases (2.1 million) were lung cancer, and 18.4% of total cancer-related deaths (1.8 million) were caused by lung cancers [27]. Lung cancer is divided into small cell lung cancer (SCLC) (15%) and non-small cell lung cancer (NSCLC) (85%), with 40% of NSCLCs being adenocarcinomas [28]. Increasing evidence links circRNAs to many processes in the development of lung cancer [29,30,31]. However, more detailed information about circRNA expression profiles, and pipelines for generating and validating hypotheses about their functions are required to deepen our understanding about the importance and molecular mechanism of circRNAs in cancers.

For the accurate and transcriptome-wide identification of circRNAs, deep RNA sequencing (RNA-seq) approaches which comprehensively cover the circRNA spectrum need to be employed. Since circRNAs are not poly-adenylated (poly-A), they are often strongly depleted from transcriptome sequences based on poly-A enrichment. In contrast, preparing sequencing libraries with rRNA depletion retains circRNAs for RNA-seq in the next step. Hence, we sequenced rRNA-depleted RNAs from 60 lung cell lines (57 lung cancer cell lines and 3 non-transformed lung cell lines) in replicates generating 3.8 billion reads in total, including 2.8 million backsplicing reads quantifying 148,811 circRNAs derived from 12,251 genes [29].

Here, we created the online database circ2GO to provide easy access to this large dataset. The integration of a broad spectrum of important orthogonal data and unique search and prediction options will foster and enhance circRNA research by providing hypotheses for pathways and miRNA targets linked to a circRNA. Mapping circRNAs to the genes in relation to all splice isoforms provides an important overview on how circularization can impact gene function and will also raise awareness for the many different circRNAs that can be derived from the same gene as well as their connection to different linear isoforms.

## 2. Results

### 2.1. Data Collection and Database Content

**Figure 1:**
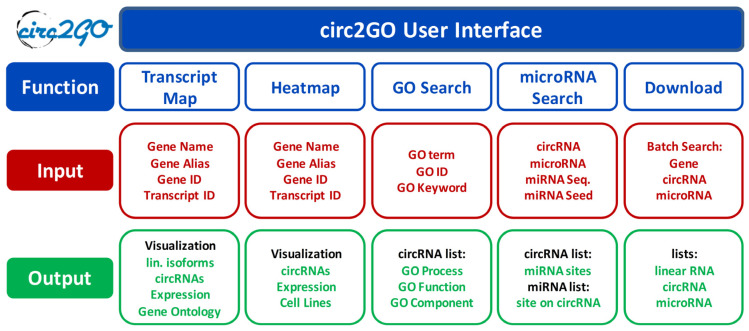
The structure of the circ2GO database. Five modules are implemented as user interfaces within circ2GO, offering multiple functionalities (blue) and their input (red) and output (green): (1) Visualization of all linear and circular isoforms for a gene including their cumulative expression in the *Transcript Map* as well as additional information; (2) Visualization of the expression of all circular RNA (circRNA) isoforms in the individual cell lines in a *Heatmap*; (3) *GO search* enables the searching for all genes and their circRNAs linked to a biological process, molecular function or cellular component; (4) *microRNA search* enables the searching for all microRNAs with binding sites on a circRNA, or all circRNA harboring binding sites for a microRNA; (5) *Download* options for all categories.

The circRNA dataset was obtained by the sequencing of rRNA-depleted RNAs from 60 lung cell lines (consisting of 50 adenocarcinoma cell lines, 7 other NSCLC cell lines and 3 non-transformed cell lines) with a total of 175 replicates. A total of 148,811 circular RNAs were detected from 12,251 genes. Each entry in the database contains a circRNA name, position, transcript of origin, expression level, gene symbol, GO annotations, and miRNAs with predicted binding sites within the circRNA. The design of the circ2GO website is intuitive and user-friendly. Generally, users can search for, obtain, and visualize information for individual circRNAs, or all circRNAs derived from a specific gene. They can search for all circRNAs derived from genes which are linked to a specified molecular function, biological process, or cellular component (GO). Lastly, they can also search for all circRNAs harboring a binding site for a specified miRNA, or vice versa. These comprehensive search options, visualization features for transcript maps and expression heatmaps, batch analyses, and download options present valuable information to the user (Figure 1).

### 2.2. circRNA Transcript Map Visualization

**Figure 2:**
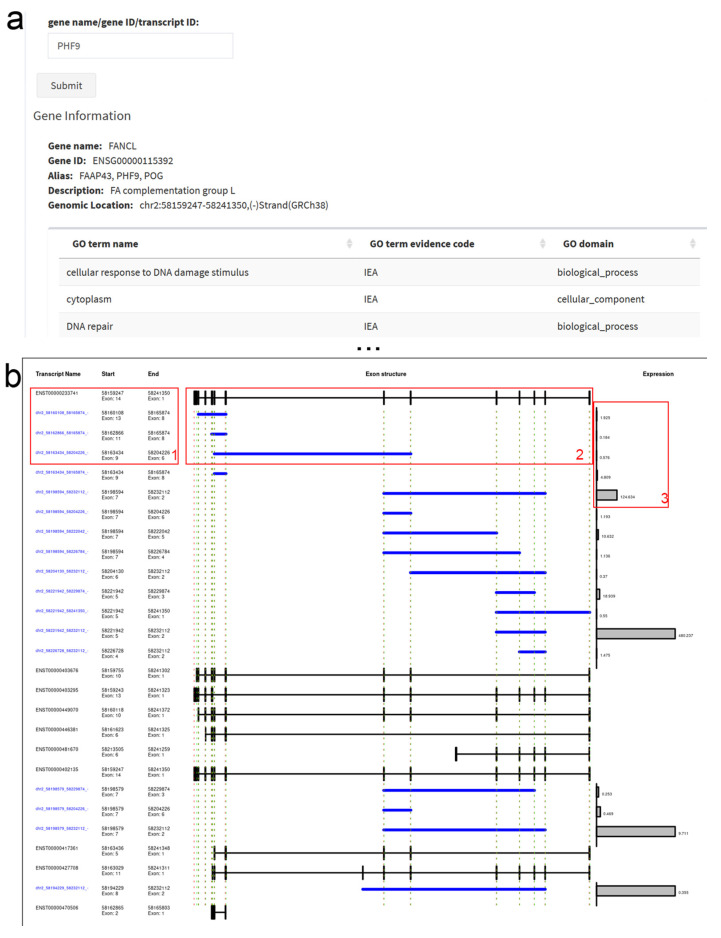
circRNA Transcript Map. (**a**) The search input accepts the gene name, alias or circRNA ID; the gene information box contains further information including GO (gene ontology) terms. (**b**) The Transcript Map shows transcript (black) and circRNA (blue) IDs and coordinates (1), the linear exons and introns as well as the circRNAs (2), and the total circRNA read counts in all cell lines (3). The dashed lines indicate exon starts and ends mapped from the first linear to the circular RNAs.

Uniquely, circ2GO includes the transcript map as a visualization module that depicts the position and abundance of all circRNAs derived from one gene and its relation to all known transcripts. The circRNA transcript map panel allows users to gain more detailed information on the circRNA position in relation to all transcripts of a queried gene, as well as their absolute abundance (Figure 2a). The map provides a precise alignment of all circRNAs and transcripts at the exon level (Figure 2b). Vertical green and red lines on the map mark the start and end of the exons, respectively. A bar diagram on the right depicts the circRNA expression profiles, allowing a rapid assessment of the relative abundance of the different circRNAs in this gene locus. Apart from the circRNA visualization, an information card displays the gene ID, gene name, gene aliases, description of the gene and genomic location. Moreover, a list of GO terms for the queried gene is provided, giving an overview of linked molecular functions, biological processes, or cellular components. Additionally, by clicking on a circRNA ID on the circRNA transcript map, a heatmap and an additional scatter plot for the selected circRNA is plotted in the circRNA heatmap panel, illustrating its expression throughout the 60 cell line panel.

### 2.3. circRNA Heatmap Visualization

**Figure 3:**
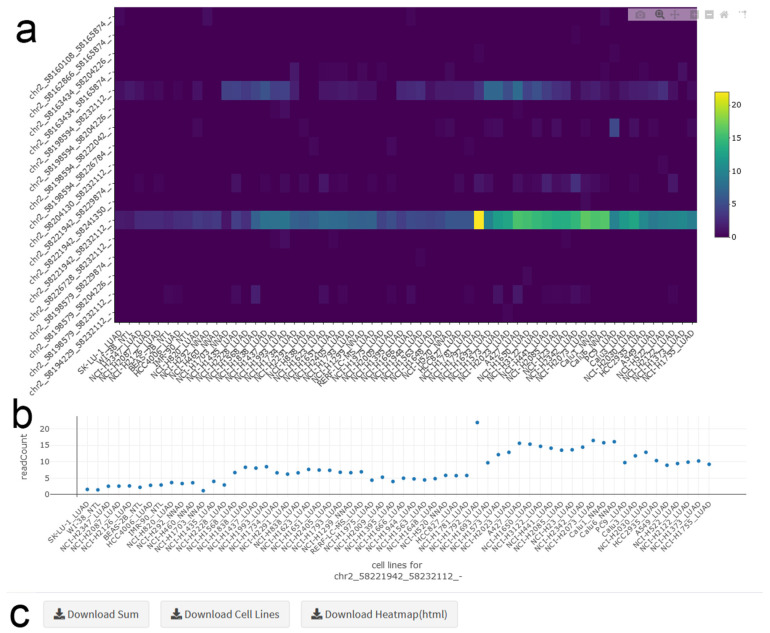
circRNA Heatmap. (**a**) The heatmap shows the abundance of all circRNAs of a selected gene in all cell lines. Hovering over the map provides details for datapoints. LUAD: Lung Adenocarcinoma, NNAD: NSCLC (non-small cell lung cancer) Non-Adenocarcinoma, NTL: Non-Transformed Lung. (**b**) The scatter plot depicts read counts for one specific circRNA in all cell lines. (**c**) Panels for downloading data and figures.

The circRNA heatmap depicts the circRNA read counts in each cell line. The expression profile for the gene of interest can be viewed either as a heatmap (multiple circRNAs included) (Figure 3a), or as a classical scatter plot (only one circRNA included) (Figure 3b). Both plots display the same order of cell lines, and the order of circRNAs matches the order in the transcript map. Heatmap representation is generated through clustering with a complete linkage algorithm. The scale bar for the heatmap shows the abundance level of the circRNAs. Hovering over the heatmap gives the read count value (normalized to library size) and the name of the cell line. In addition to displaying the expression profile in plots, the average circRNA expression for each cell line, as well as the total circRNA counts can be downloaded.

### 2.4. Gene Ontology Search

Gene Ontology (GO) is an important bioinformatics project that aims to uniformly define the representation of gene characteristics and gene products in all species. The main uses of GO are retrieving functional profiles of gene sets by performing enrichment analyses, as well as GO term annotation of individual genes in the categories of molecular function, biological process, and cellular component. All GO terms are listed for the gene of interest in the transcript map section.

As a unique feature, circ2GO offers a reverse search, i.e., the option to search for all circRNAs derived from genes involved in a specific molecular function, biological process, or cellular component via the “GO Search” module in circ2GO. Users can find circRNAs by GO terms and download the data with circRNA expression profiles for further functional exploration of circRNAs.

**Figure 4:**
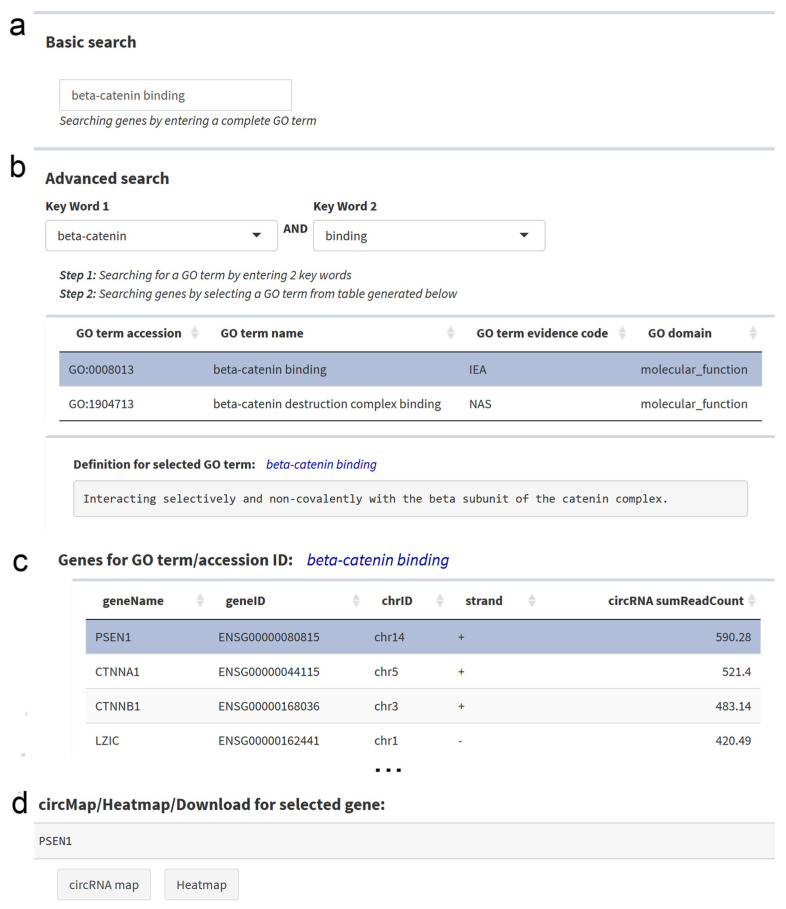
Gene Ontology (GO) Search Panel. (**a**) The basic search option allows for the search of GO terms, GO IDs or keywords. (**b**) The advanced search option allows for the combination of keywords from lists of keywords in GO terms. By selecting one row in the search results, the annotation for the respective GO term is shown below. (**c**) A table lists genes associated with the respective GO term, as well as the total abundance of the circRNAs derived from each gene in all cell lines. (**d**) At the bottom, direct links to the circRNA transcript map and the circRNA heatmap are provided for the selected gene.

The basic search option can be applied for a GO ID, a complete GO term, or a part of it (Figure 4a). The advanced search option allows the combination of keywords from GO terms, and then the selection of a specific GO term from the resulting list (GO accession, GO name, GO evidence code, GO domain) (Figure 4b). For both search options, all genes with the same GO term are listed in an interactive table providing the respective gene IDs and the total circRNA expression from this gene. The search results can be downloaded as a csv file. By selecting one gene in the table, a circRNA transcript map and heatmap for all circRNAs of this gene can be obtained.

### 2.5. circRNA–miRNA Search

MicroRNAs (miRNAs) are small, single-stranded and highly-conserved non-coding RNA molecules, which can bind to target mRNAs and silence their protein expression by mRNA destabilization or translational inhibition [32,33]. Circular RNAs can function as molecular sponges by binding to miRNAs, with the most prominent example being CDR1-AS (CDR1 Antisense RNA) [34]. MiRNA expression levels in tumors may be altered by circRNAs, which implies that miRNA–circRNA networks may be involved in the development of cancer [35,36,37].

Hence, we included a prediction of miRNA binding sites within circRNAs into circ2GO. For this circRNA–miRNA dataset, 25,166 highly abundant circRNAs from 6578 genes were filtered with the threshold of at least 2 reads (read count normalized) in any cell line. 897 high-confidence human miRNAs were downloaded from miRBase (http://www.mirbase.org) [38]. The prediction for circRNA–miRNA binding sites was performed by using TargetScan [39] and miRanda [40].

**Figure 5:**
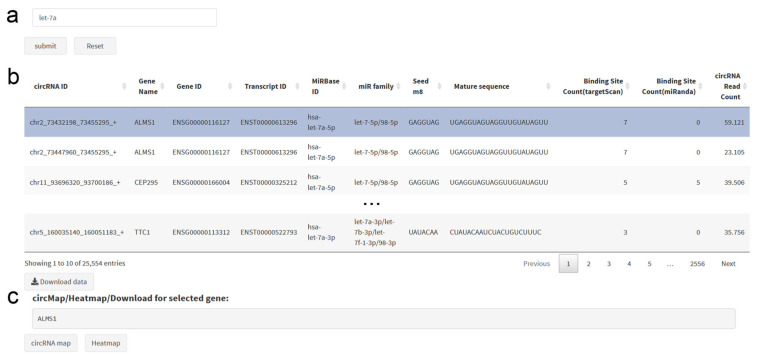
circRNA–microRNA Search Panel. (**a**) The search can be performed for gene name, gene ID, circRNA ID, microRNA name, miRBase ID, microRNA sequence or microRNA seed sequence. (**b**) The results table lists pairs of circRNAs and microRNAs, which can also be downloaded. (**c**) At the bottom, direct links to the circRNA transcript map and the circRNA heatmap are provided for the selected gene.

The circRNA–miRNA search tab allows the search either for all circRNAs targeting a specified miRNA, miRNA family or miRNA seed region sequence, or for all miRNAs with binding sites in a specified circRNA, or in all circRNAs of a specified gene (Figure 5). Approximate string matching is supported for all of the aforementioned search criteria, allowing fuzzy inputs. The resulting dataset provides detailed information for circRNA–miRNA pairs, including circRNA expression and binding site counts, allowing the identification of circRNA–miRNA pairs with high circRNA abundance and multiple binding sites. For a selected gene, a circRNA transcript map and circRNA heatmap can be obtained with one click. The search results can be downloaded as a csv file.

### 2.6. Data Download

This batch download option enables users to easily transfer data for further individual analysis. The users can download the circRNA dataset completely or partially, by selecting cell line names, genes, or miRNAs by multi-line text input with cell line, circRNA, miRNA, gene symbol or miRNA sequence. Approximate string matching is supported. The circRNA dataset is formatted into two different levels: (1) the gene level which contains an aggregation of all circRNAs of one gene; (2) the backsplice level which contains all individual circRNAs separately. The circRNA–miRNA database is also available for download.

In three ways, the presented data have been selectively validated: (1) The existence of selected circRNAs identified by RNA-seq has been experimentally validated by RT-PCR and RT-qPCR in our previous study [29]. (2) We have selected five circRNA–miRNA pairs from the literature and also found all five of the following associations in circ2GO: circCDR1-AS: miR-7 [34,41], circCDR1-AS: miR-671 [41], circMTO1: miR-9 [35], circHIPK3: miR-124 [4], circHIPK3: miR-30a-3p [42]. (3) We tested whether examples of pairs of circRNA genes and GO terms from circ2GO could be experimentally validated in the literature and found, e.g., circZNF609 linked to regulation of myoblast differentiation and myogenesis [9] as well as circSHPRH linked to protein ubiquitination [43].

## 3. Methods and Software

The circRNA dataset contained within circ2GO was derived from RNA-seq data. Libraries for RNA sequencing were prepared by depleting ribosomal RNA. Raw reads were mapped using *Tophat2* [44] with parameters set as *-a 6 -m 2 -g 1 -p 16*. Unmapped reads were extracted as a new bam file and were then mapped again to the reference genome with the *TopHat-Fusion* module (included in TopHat2). *CIRCexplorer2* [45] was used to process bam files and obtain the list of circRNAs with standard parameters. The circRNA expression level was calculated by the number of reads that were mapped to a backsplice site. All reads were mapped to the Ensembl GRCh38 gene set in the steps above. In total, 148,811 circular RNAs originating from 12,251 genes were detected and quantified.

The GO annotations were downloaded from the Ensembl BioMart [46]. The GO annotation dataset was integrated with our circRNA dataset according to gene ID, with version suffixes removed.

*DESeq2* [47] was utilized for circRNA read count normalization across all samples (*n* = 175). A total of 25,166 highly abundant circRNAs from 6578 genes were filtered with the threshold of at least 2 reads (read count normalized) in any cell line. With the bed file based on the circRNA coordinates and strand, *getfasta* was used to obtain sequences for the circRNA exons. All exons within the span of the circRNA splice sites were included. Pieces of exonic sequences were concatenated sequentially to generate a complete circRNA sequence. A total of 897 high-confidence human miRNAs were downloaded from miRBase [38]. miRNA–circRNA interactions were predicted by miRanda [48] and TargetScan (Release 7.2) [39], respectively.

circ2GO was implemented using HTML and in R language (v3.6.0) [49] with shiny package. The *Shiny* application was built with RStudio [50]. Part of the interface component consists of web pages that were designed and implemented in HTML/CSS. The code is available on GitHub at https://github.com/airbox11/circ2GO.

## 4. Availability

The circ2GO database is freely and without registration available at https://circ2GO.dkfz.de.

## 5. Conclusions

The functions of circRNAs are gaining considerable interest across many areas of life sciences and have become a key focus in cancer research. To date, thousands of circRNAs have been detected in various species and tissues. While several functions have been proposed for these circRNAs, our understanding of their precise biological roles and significance is still limited for the vast majority of circRNAs. With circ2GO, we present a comprehensive database for human circRNAs, including their expression in a broad cell line panel, their associated GO terms regarding molecular functions, biological processes, and cellular components, as well as comprised miRNA binding sites predicted by two independent algorithms. Visualizations in transcript maps and heatmaps, advanced forward and reverse search options, batch search and download options, combined with its intuitive and easy use will make circ2GO a valuable tool for circRNA research.

We imagine that the most widespread applications of circ2GO will be: (1) the comparison of all circRNAs for a given gene or transcript, including their expression levels; (2) the search for cell lines with a particularly high or low expression of a specific circRNA; (3) the search for GO terms associated with a circRNA by virtue of its linear counterpart to form hypotheses about its potential impact on the functions of pathways to be experimentally tested; (4) the search for all circRNAs derived from genes involved in a specific molecular function, biological process, or cellular component of interest; (5) the search for abundant circRNAs harboring binding sites for a particular miRNA of interest, and their prioritization based on their expression and number of binding sites; (6) the search for all binding sites of high confidence within a specific circRNA of interest to form hypotheses about its potential function as ceRNA (competing endogenous RNA) to be experimentally tested.

While the expression data provided in circ2GO are limited to lung-derived cell lines, other functions of circ2GO are not restricted to lung cancer or cancer research in general, but can be applied to other areas of human life sciences. The “GO Search” tool and the “microRNA search” tool can also be applied if the same circRNA has been identified in any other context. While the expression patterns are derived from a broad panel of human lung cell lines, the sequencing depth as well as the rRNA depletion (instead of polyA-enrichment) of our underlying transcriptomic study gives a comprehensive picture of the landscape of human circRNAs. For comparison, circ2GO includes 148,811 distinct circRNAs, while human studies stored in one of the leading circRNA databases, circBase, add up to only 92,375 circRNAs. Hence, circ2GO provides a map of human circRNAs with a large depth. Moreover, the transcript map visualization, the “GO Search” options, and the “microRNA Search” options are fully separate of the underlying expression dataset, and can therefore be universally applied, independent of the user’s research area for this large set of circRNAs.
